# The Adaptive Mechanism of Ginseng Rhizomes in Response to Habitat Changes

**DOI:** 10.3390/cimb46110728

**Published:** 2024-10-30

**Authors:** Meng Zhang, Yingxin Sun, Zeliang Lv, Hongmei Lin, Mei Han, Limin Yang

**Affiliations:** Co-constructing Key Laboratory by Province and the Ministry of Science and Technology of Ecological Restoration and Ecosystem Management, College of Chinese Medicinal Material, Jilin Agricultural University, Changchun 130118, China; zhangmeng@mails.jlau.edu.cn (M.Z.);

**Keywords:** *Panax ginseng*, rhizome, habitat change, phenotypic plasticity, lignin, endogenous hormone

## Abstract

*Panax ginseng*, a perennial medicinal plant, utilizes its dried roots and rhizomes for medicinal purposes. Currently, in China, ginseng cultivation employs two methods: under-forest and farmland planting. These methods create distinct habitats, significantly influencing the ginseng’s rhizome morphology and, consequently, its economic value. In this study, two-year-old ginsengs were transplanted into farmland (TCG), a larch forest (TLCG) and a *Quercus mongolica* forest (TQCG) to analyze the differences in rhizome phenotypes caused by habitat changes. The results showed that there were significant differences in light intensity and the soil’s available phosphorus and potassium contents between farmland and forest environments. The differences in habitats led to different adaptability of the ginseng’s rhizome morphology. Compared with TCG, the rhizomes of TLCG and TQCG were significantly elongated by 48.36% and 67.34%, respectively. After the rhizomes’ elongation in TLCG and TQCG, there was an increase in indole-3-acetic acid (IAA) contents and a decrease in lignin contents. By analyzing the expression of key genes, we found that, compared with TCG, the expression of key enzymes of lignin biosynthesis genes such as *PgCOMT* and *PgCCR4* were down-regulated. The difference in ginseng’s rhizome length is related to the signal transduction pathway of auxin and gibberellin. In addition, we preliminarily screened out transcription factors *PgWRKY75*, *PgDIV,* and *PgbHLH93.1*, which can actively respond to habitat changes and play important roles in the elongation of ginseng rhizomes. In summary, this study elucidates the phenotypic plasticity strategy of ginseng rhizomes in response to habitat changes and delineates the regulatory mechanism behind phenotypic adaptation, offering novel insights into ginseng’s morphogenesis.

## 1. Introduction

*Panax ginseng*, a perennial herb in the genus Panax within the family Araliaceae, is highly esteemed as the “king of herbs” due to its significant medicinal and economic value. Its dried roots and rhizomes are utilized in traditional medicine [1,2,3]. Currently, ginseng is categorized into wild ginseng, mountain-cultivated ginseng, and cultivated ginseng types based on cultivation practices and growth environments. Wild ginseng, known for its superior quality, is now nearly extinct [4,5]. Mountain-cultivated ginseng, cultivated in environments that mimic the natural habitat of wild ginseng, experiences minimal human intervention and a prolonged growth cycle. This method not only preserves some morphological and qualitative characteristics of wild ginseng but also elevates its market value significantly, often reaching ten to several dozen times that of cultivated ginseng [6,7]. Conversely, cultivated ginseng is grown in a crop-like manner, often with practices aimed at maximizing yield, such as high-density planting, the extensive use of water and fertilizers, and excessive pesticide application. These practices detract from the aesthetic and qualitative attributes typified by wild ginseng, leading to a marked difference in appearance and internal quality [8]. Notably, wild ginseng and mountain-cultivated ginseng exhibit long, tortuous rhizomes, in contrast to the short rhizomes of cultivated ginseng. Rhizome length is a critical indicator of ginseng’s “excellent shape” and plays a vital role in quality assessment [9]. These observations suggest that habitat changes significantly influence ginseng’s phenotypic plasticity, implying that rhizome phenotype alterations could be an adaptive growth strategy in response to environmental variations, which may also indicate shifts in internal quality. However, the key ecological factors driving these phenotypic changes in ginseng roots remain to be elucidated.

The interplay between plants and their environments represents a foundational topic in ecological research, with plant functional traits being pivotal in how plants respond to habitat alterations [10]. Phenotypic plasticity is deemed a critical attribute in plants’ adaptation to varying growth conditions or external stimuli. Originally, Bradshaw defined phenotypic plasticity as the capacity of a single genotype to exhibit diverse phenotypes in response to different environmental contexts [11]. Notably, plants’ plastic responses to varied environments are predominantly adaptive. This adaptability is crucial for plants to navigate environmental fluctuations, playing a significant role in their growth, survival, and distribution patterns [12,13,14,15]. The ginseng rhizome, an underground stem resembling a root in shape, is particularly susceptible to soil’s environmental changes due to its subterranean growth and direct soil contact [16]. Research has revealed that plant roots, though not directly exposed to light, can perceive various signals. These include the transmission of signal molecules from stem to root, roots’ direct light induction, and light conduction within the plant, all of which can initiate root photomorphogenesis and influence root phenotype alterations [17,18]. Within this complex signaling network, endogenous hormones emerge as crucial signaling molecules. They can swiftly react to environmental shifts, relay signals to downstream cells, and activate key transcription factors. This process modulates the expression of relevant genes, thereby mediating plant phenotypic changes [19,20,21]. Intriguingly, plant morphology is often determined by cell number and size, with the latter being intimately linked to cell wall elasticity. Lignin, a key component of the cell’s secondary wall, plays a vital role in defining cell wall elasticity [22]. An accumulation of lignin in the cell wall reduces its elasticity, constraining cell expansion and, consequently, influencing plant morphology [23].

Our prior research established that the phenotype of ginseng roots undergoes significant alterations across varying habitats, a phenomenon intricately linked to the lignin biosynthesis pathway and hormone signal transduction pathway [2]. In the current study, we transplanted two-year-old ginseng plants of uniform size into distinct environments: farmland, a larch forest, and a *Quercus mongolica* forest. After one growth cycle, these plants were harvested. We analyzed the differences in growth conditions and identified the principal ecological factors influencing phenotypic variations in ginseng roots. Additionally, we investigated the responses of ginsenoside content, endogenous hormone contents, and lignin concentration in the ginseng rhizome to habitat changes. The expression levels of key genes and transcription factors associated with the rhizome phenotype identified in our previous investigations were also measured. Our objective is to elucidate the strategies of phenotypic plasticity in ginseng rhizomes in response to habitat changes and to decode the internal regulatory mechanisms of these phenotypic adjustments. Understanding these processes is crucial for enabling the targeted cultivation of ginseng with “excellent shape” and for guiding its high-quality development.

## 2. Materials and Methods

### 2.1. Plant Materials

For this study, we selected two-year-old ginseng seedlings that were disease-free, had intact rhizomes, had robust and complete ginseng whiskers, and were of uniform size. In early May 2023, these seedlings were transplanted into three distinct environments: the Medicinal Botanical Garden of Jilin Agricultural University, a larch forest, and a *Quercus mongolica* forest located in Houshan (125°24′ E, 43°48′ N). Each transplantation plot accommodated 80 ginseng seedlings. The farmland environment within the medicinal botanical garden was designed to simulate open space conditions, with cultivation and farmland management practices tailored to ensure the normal growth of ginseng, akin to cultivated ginseng farming. The forest transplantation aimed to mimic the cultivation and management practices of mountain-cultivated ginseng, facilitating natural growth conditions. The ginseng seedlings from the farmland (TCG), under the larch forest (TLCG), and under the *Quercus mongolica* forest (TQCG) were harvested in mid-September of the same year. The entire ginseng plant was carefully dug out from each location and washed thoroughly. Twelve ginsengs were selected to measure their growth and development indices. A portion of the fresh samples was immediately frozen in liquid nitrogen and stored at −80 °C in an ultra-low-temperature freezer for future analysis. Another portion of the fresh samples was preserved in formaldehyde–acetic acid–ethanol (FAA) fixative solution for later preparation of paraffin sections. The remaining samples were dried in an oven at 45 °C to a constant weight for ginsenoside content analysis.

### 2.2. Information Collection and Determination of Habitat

We measured the elevation, light intensity, and soil temperature at the TCG, TLCG, and TQCG plots (9 sample sites). The elevation was recorded using a global positioning system (GPS) (eTrex 30, Garmin, Olathe, KS, USA). Light intensity at the same height and position as ginseng leaves was gauged with an illuminance meter (AS803, Dongguan Wanchuang, Dongguan, China), and soil temperature was measured with a geothermometer (HY-1, Hongxing Instrument factory of Wuqiang county, Hebei, China) from 10 am to 12 am. Additionally, we cleared the soil surface in these sample plots of any humus, such as litter. Soil samples were collected using the five-point sampling method. The collected fresh soil was placed into sealable bags, air-dried, and then sifted through a 20-mesh sieve to prepare for the analysis of the soil’s physical and chemical properties. The specific methodologies for these analyses were conducted in accordance with the guidelines provided in Bao Shidan’s “Soil Agricultural Chemistry Analysis” [24].

### 2.3. Preparation and Observation of Paraffin Section

Ginseng rhizome samples, which had been fixed in formaldehyde–acetic acid–ethanol fixative (FAA) for over 24 h, underwent a dehydration process before being embedded in paraffin. Subsequently, these embedded samples were sectioned into slices with a thickness of 10 μm. These slices were affixed to glass slides and allowed to dry at 40 °C overnight. Following the drying process, the slices were dewaxed and stained using a Saffron-O fast green dye solution [25]. Finally, the glass slides were mounted with a neutral balsam solution for sealing and subsequently examined under an optical microscope.

### 2.4. Metabolic Component Contents Determination

To quantify the ginsenoside contents in ginseng, we initiated the process by weighing 0.25 g of dried ginseng rhizome powder. This sample was then subjected to ultrasonic extraction using a methanol solution. Following extraction, the solution was filtered, and the solvent evaporated. The residue was redissolved in methanol, transferred to a 5 mL volumetric flask, and then filtered through a 0.22 µm filter in preparation for HPLC analysis. The analysis was conducted using an Agilent 1260 generation II HPLC system (Agilent, CA, USA) equipped with an autosampler. The gradient elution procedure adhered to the method established by Zhang [2]. To create a standard curve, we prepared a methanol aqueous solution containing specific ginsenosides (Rg1, Re, Rf, Rb1, Rc, Rb2, Rb3, and Rd) and diluted it to the appropriate concentration. The standard regression equations for each ginsenoside were as follows: Rg1: Y = 471.1X + 7.3252 (R^2^ = 0.9999); Re: Y = 385.02X + 5.7274 (R^2^ = 0.9997); Rf: Y = 357.2X + 0.9383 (R^2^ = 0.9991);Rb1: Y = 276.14X + 5.6388 (R^2^ = 1); Rc: Y = 239.24X + 5.5644 (R^2^ = 0.9999); Rb2: Y = 269.98X + 3.0672 (R^2^ = 0.9999); Rb3:Y = 269.55X + 0.7054 (R^2^ = 0.9994); and Rd: Y = 286.91X + 0.9281 (R^2^ = 0.9992). Using the above equations, we calculated the concentrations of eight monomeric saponins in the ginseng rhizome. Additionally, the method for determining the total saponin content in ginseng followed Zhang’s protocol [2].

To determine the lignin contents in ginseng, we employed the acetyl bromide method to determine the fresh taproot samples of the ginsengs. This technique involves the acetylation of phenolic hydroxyl groups in lignin using an acetyl bromide–glacial acetic acid mixed solution. The lignin content is then quantified based on the characteristic maximum ultraviolet absorption peak of the acetylated lignin solution at 280 nm [26]. We followed the specific operational procedures as outlined in the lignin content detection kit (BC4205) (Beijing Solarbio Science & Technology Co., Ltd., Beijing, China).

To quantify the phytohormone contents in ginseng, we started by weighing 0.10 g of the fresh ginseng rhizomes. To this, we added 0.90 mL of precooled phosphate-buffered solution (PBS) (0.1 M, pH 7.4) and proceeded to homogenize the mixture on ice. The homogenate was then centrifuged at 3000 rpm for 20 min, and the resulting supernatant served as the hormone extract. The contents of plant hormones within the ginseng rhizomes, specifically indole-3-acetic acid (IAA), gibberellic acid (GA), zeatin (ZT), kinetin (KT), salicylic acid (SA), and abscisic acid (ABA), were determined using a plant hormone ELISA detection kit (Shanghai Enzyme-linked Biotechnology Co., Ltd., Shanghai, China) [27]. The absorbance (OD) was measured at a wavelength of 450 nm using an enzyme-labeled instrument, and the concentrations of the plant hormones in the samples were calculated based on a standard curve.

### 2.5. Determination of Key Genes’ Expression

qRT-PCR technology was used to determine the expression of genes related to the difference in ginseng rhizomes’ lengths in different habitats. The transcriptome data of ginseng rhizome samples in different cultivation environments were obtained by sequencing. Differentially expressed genes were screened according to *p* < 0.05 and |log2-fold change| ≥ 1 as the significance threshold. Differentially expressed genes were annotated and significantly concentrated according to the KEGG (http://www.genome.jp/kegg/ accessed on 24 November 2023) databases (unpublished data from our research group). The total RNA were extracted from fresh ginseng rhizome samples using a kit specifically designed for plants rich in polysaccharides and polyphenols. The extracted RNA, upon meeting quality standards, was reverse transcribed into cDNA using the Prime Script II 1st cDNA Synthesis Kit (Takara Bio Inc., Dalian, China), which then served as the template for further analysis. Key genes implicated in lignin biosynthesis and the signal transduction of endogenous hormones, identified through previous studies, were selected for examination. The *PgGAPDH* gene was utilized as the reference gene for normalization purposes. Specific primers for these genes were designed using Primer 5.0 software (Table S1), facilitating the execution of real-time fluorescence PCR assays. The PCR reaction mixture comprised 7 µL of sterilized water, 10 µL of SYBR^®^ Premix Ex TaqTM (Takara Bio Inc., Dalian, China), 1 µL of each primer, and 1 µL of the cDNA template, totaling 20 µL [28]. The PCR cycling conditions were set as follows: an initial pre-denaturation at 95 °C for 30 s, followed by 40 cycles of denaturation at 95 °C for 5 s, annealing at 55 °C for 30 s, and extension at 72 °C for 20 s. Gene expression levels were quantified using the 2^−ΔΔCt^ method [29].

### 2.6. Statistical Analysis

The raw data collected during this study were organized and compiled using MS Excel 2021 (Microsoft, Redmond, WA, USA). For the statistical analysis of the data, we employed SPSS software, version 19.0 (IBM, Armonk, NY, USA). The Duncan test was used to test mean comparisons. One-way analysis of variance (ANOVA) was conducted for significance analysis. The confidence interval was set to 95%, and *p* < 0.05 was chosen to indicate the significant differences between the treatment groups. Bioinformatic analyses were executed using tools available on MetWare (http://www.metware.cn/, accessed on 24 November 2023). For the creation of graphical illustrations that effectively communicate our experimental results, GraphPad Prism version 10.1 (GraphPad Software Inc., San Diego, CA, USA) was utilized.

## 3. Results

### 3.1. Analysis of the Difference in Ecological Factors in Ginseng Land

**Figure 1:**
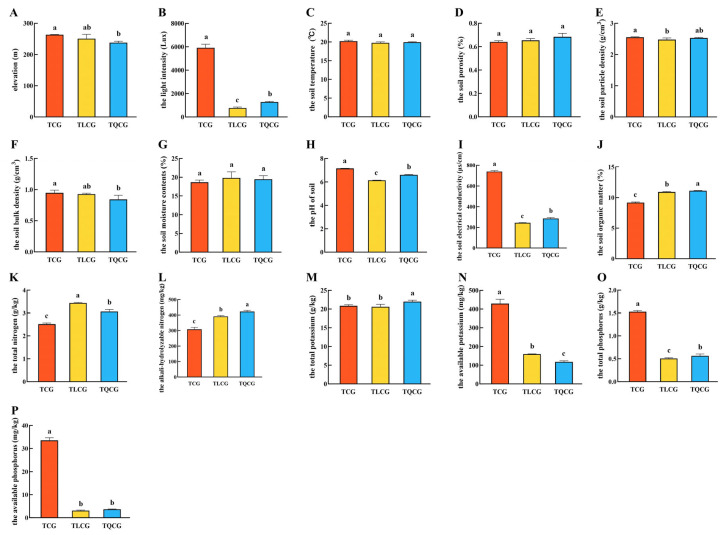
Ecological factor indexes of ginseng in various plots. (**A**): elevation; (**B**): the light intensity; (**C**): the soil temperature; (**D**): the soil porosity; (**E**): the soil particle density; (**F**): the soil bulk density; (**G**): the soil moisture contents; (**H**): the pH of soil; (**I**): the soil electrical conductivity; (**J**): the soil organic matter; (**K**): the total nitrogen; (**L**): the alkali-hydrolyzable nitrogen; (**M**): the total potassium; (**N**): the available potassium; (**O**): the total phosphorus; (**P**): the available phosphorus. Different lowercase letters indicate significant differences at the level of *p* < 0.05. Error bars depict standard deviations (±SD).

The growth environment significantly influences plant phenotype, with light, temperature, moisture, air, and soil identified as the primary ecological factors [30,31,32]. In our study, two-year-old ginseng seedlings of uniform size were transplanted into farmlands (TCG), larch forests (TLCG), and *Quercus mongolica* forests (TQCG). Our observations revealed no marked differences in elevation or soil temperature among the three sites (Figure 1A,C). However, the light intensity of the TLCG and TQCG plots was significantly lower than that of the TCG plots (Figure 1B). Despite constructing a shade shed during cultivation, light intensity remained substantially higher in the TCG plot compared to the dense forest environments of the TLCG and TQCG, with differences ranging from approximately 4.6 to 7.8 times. The direct contact of ginseng rhizomes with the soil underscores the dependency of their growth and development on soil conditions. Our analysis indicated no significant differences in the soils’ physical properties—such as moisture content, porosity, particle density, and bulk density—between the TLCG and TQCG plots compared to the TCG plot (Figure 1D–G).

Nitrogen, phosphorus, and potassium are essential nutrients for plant growth and development. In our study, the TCG plots exhibited the highest total phosphorus content at 1.53 g/kg (Figure 1O), which met the second-class soil standard (Table S2) and was 203.13% and 170.60% higher than TLCG and TQCG, respectively. Conversely, the levels of organic matter, total nitrogen, and total potassium in the TCG plot were 9.17%, 2.51 g/kg, and 20.84 g/kg, respectively, which were significantly lower than those in the TLCG and TQCG (Figure 1J,K,M). Despite these different values, the organic matter, total nitrogen, and total potassium contents of the three plots all met the first-class soil standard (Table S2). Interestingly, alkali-hydrolyzable nitrogen, available potassium, and available phosphorus represent readily available nutrients that plants can directly absorb and utilize, significantly influencing plant growth and development [33]. The alkali-hydrolyzable nitrogen content in all three plots exceeded 308.00 mg/kg (Figure 1L), aligning with the first-class soil standard (Table S2). The TCG plot had the highest contents of available potassium and phosphorus, which were 5.83 to 7.92 times and 9.23 to 11.06 times those of the TLCG and TQCG plots, respectively (Figure 1N,P). However, it is crucial to note that the available phosphorus contents of the TLCG and TQCG plots were exceptionally low, at only 3.03 mg/kg and 3.63 mg/kg, respectively, which only met the fifth-class soil standard (Table S2), while the available phosphorus content of the TCG plot was 33.53 mg/kg, which met the second-class soil standard (Table S2). It can be seen that the growth environments of the TLCG and TQCG plots are similar to each other, but compared with the TCG plot, there are obvious differences in light intensity and soil nutrients in the farmland and understory.

### 3.2. Analysis of Growth and Development Differences of Ginseng Rhizomes in Different Habitats

**Figure 2:**
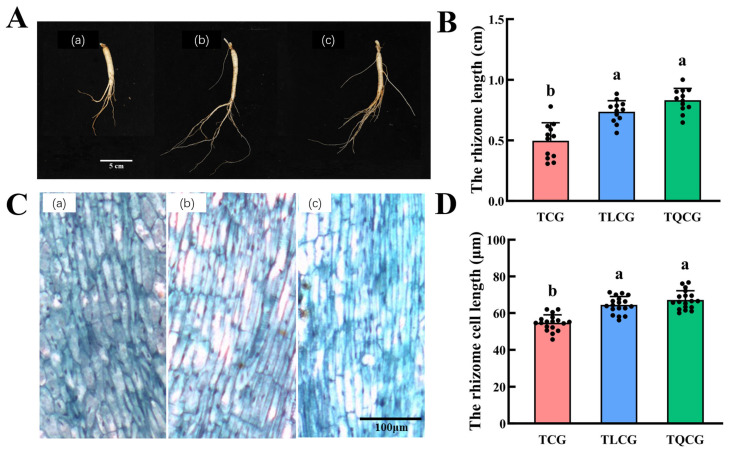
Growth index and microstructure of ginseng rhizomes in longitudinal sections. (**A**): root morphology of ginsengs (scale = 5 cm); (**B**): ginseng rhizome length; (**C**): microstructure of cork layer cells of ginseng rhizomes (scale = 100 μm); (**D**): cell length of cork layer of ginseng rhizomes. (**a**): TCG; (**b**): TLCG; (**c**): TQCG. Different lowercase letters indicate significant differences at the level of *p* < 0.05. Error bars depict standard deviations (±SD).

In our study, ginseng seedlings of uniform size were transplanted into various habitats, resulting in observable changes in rhizome phenotypes across the TCG, TLCG, and TQCG plots (Figure 2B). The rhizome length in the TCG plot was the shortest, measuring only 0.50 cm. In contrast, the rhizomes in the TLCG and TQCG plots exhibited lengths that were 48.36% and 67.34% longer than those in the TCG plot, respectively. This variation indicates that ginseng rhizome length adapts differently to habitat changes. To gain a deeper insight into the rhizome phenotype, we examined the microstructure of longitudinal sections of rhizomes from the TCG, TLCG, and TQCG plots using paraffin sections (Figure 2C). The cell arrangement in the TLCG and TQCG rhizomes was observed to be more compact and orderly compared to that in the TCG rhizomes. Additionally, measurements of rhizome cell lengths revealed that cells in the TCG rhizomes were the shortest, while those in the TLCG and TQCG rhizomes were significantly longer (Figure 2D). This finding aligns with the observed differences in rhizome phenotypes among the plots, suggesting that the length of ginseng rhizomes of the same age is intrinsically linked to the elongation of individual cells.

### 3.3. Analysis of Difference in Metabolic Components’ Accumulation of Ginseng in Different Habitats

#### 3.3.1. Changes in Ginsenoside Content in Ginseng

**Figure 3:**
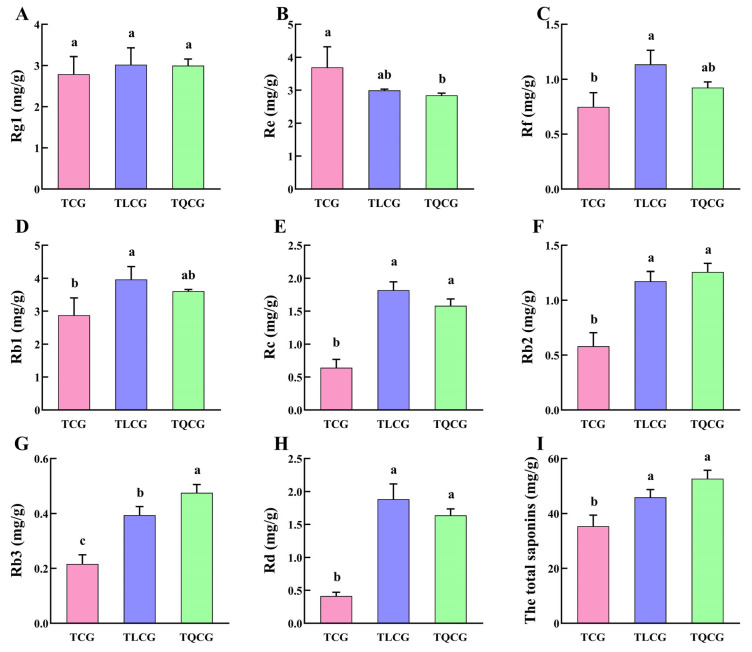
Ginsenoside contents in different habitats. (**A**): Rg1; (**B**): Re; (**C**): Rf; (**D**): Rb1; (**E**): Rc; (**F**): Rb2; (**G**): Rb3; (**H**): Rd; (**I**): the total saponins. Different lowercase letters indicate significant differences at the level of *p* < 0.05. Error bars depict standard deviations (±SD).

Ginsenosides, the primary bioactive compounds in ginseng, serve as secondary metabolites that dynamically respond to habitat changes [34]. In our study, we analyzed the content variations of eight monomeric saponins (Rg1, Re, Rf, Rb1, Rb2, Rb3, Rc, Rd) and the total saponins in the rhizomes from the TCG, TLCG, and TQCG plots (Figure 3). The findings revealed that monomeric saponins, particularly the protopanaxadiol saponins, exhibited significant responsiveness to habitat alterations. Specifically, the contents of Rb1, Rb2, Rb3, Rc, and Rd in the TLCG and TQCG rhizomes were notably higher than those in the TCG rhizomes. Moreover, the total saponin content in both the TLCG and TQCG plots was significantly elevated, showing increases of 29.90% and 49.03%, respectively, compared to the TCG. These results underscore that ginseng’s adaptation to changes in habitat involves not only alterations in rhizome phenotype but also significant shifts in internal quality. This demonstrates the interconnectedness of external phenotypic characteristics and internal quality in ginseng’s response to environmental variations.

#### 3.3.2. Changes in Lignin Content in Ginseng

**Figure 4:**
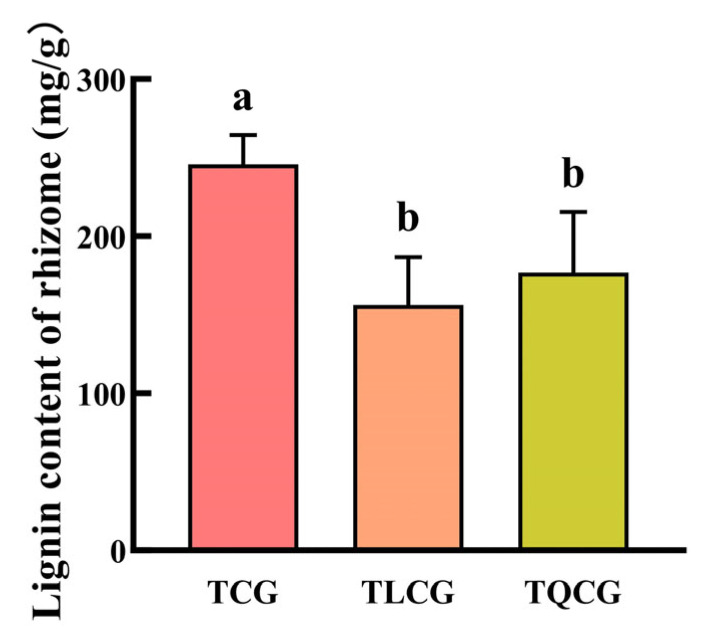
Lignin contents of ginseng in different habitats. Different lowercase letters indicate significant differences at the level of *p* < 0.05. Error bars depict standard deviations (±SD).

Lignin, a complex organic polymer, is classified among secondary metabolites and predominantly located within plant cell walls. It plays a crucial role in reinforcing mechanical support and stability, contributing to a plant’s adaptability and resilience to environmental stresses [23]. In our study, lignin content was measured in the rhizomes from the TCG, TLCG, and TQCG plots (Figure 4). The results indicated that the TCG rhizomes had the highest lignin content at 245.43 mg/g, significantly surpassing those of the TLCG and TQCG by 57.47% and 38.86%, respectively. However, the difference in lignin content between the TLCG and TQCG plots was not statistically significant. This pattern is in stark contrast to the observed rhizome phenotypes among the three habitats. This result shows that the growth of ginseng rhizomes was limited, which may be related to the lignification of rhizome cell walls caused by excessive lignin deposition.

#### 3.3.3. Changes in Endogenous Hormone Content in Ginseng

**Figure 5:**
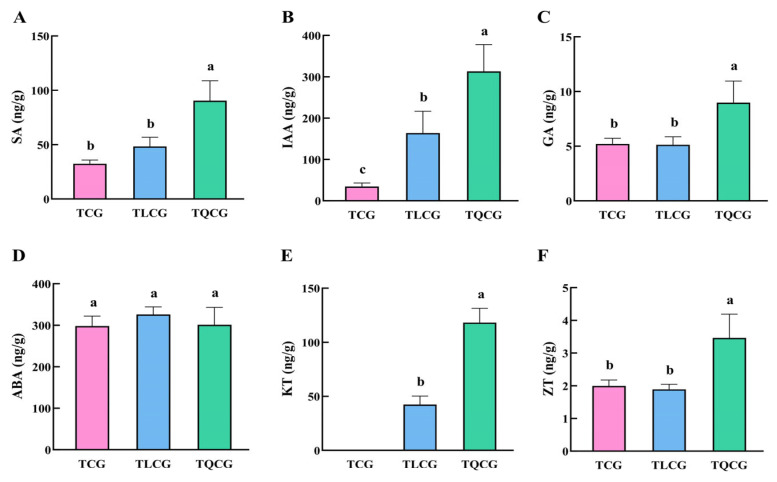
Endogenous hormone contents of ginseng in different habitats. (**A**): Salicylic acid (SA); (**B**): indole-3-acetic acid (IAA); (**C**): gibberellin (GA); (**D**): abscisic acid (ABA); (**E**): kinetin (KT); (**F**): zeatin (ZT). Different lowercase letters indicate significant differences at the level of *p* < 0.05. Error bars depict standard deviations (±SD).

Endogenous hormones are crucial in regulating plant growth and development [35,36]. In our study, we analyzed the contents of various endogenous hormones affecting rhizome growth in ginseng from the TCG, TLCG, and TQCG plots, including salicylic acid (SA), auxin (IAA), gibberellin (GA), abscisic acid (ABA), kinetin (KT), and zeatin (ZT) (Figure 5). The results showed that, except ABA, the other hormone contents were significantly different in the rhizomes of the TCG and TQCG. KT was not detected in the TCG, but the contents in the TLCG and TQCG were 42.41 ng/g and 118.14 ng/g, respectively (Figure 5E). The contents of SA, GA, and ZT in TQCG rhizomes were significantly higher than those in the TCG, which were significantly increased by 179.06%, 72.72%, and 73.41%, respectively. However, there was no significant difference between the TLCG and TCG (Figure 5A,C,F). It is worth noting that, compared with the TCG, the IAA concentration in the TLCG and TQCG increased by 372.36% and 803.01%, respectively (Figure 5B). This correlation is similar to the observed change in rhizome length, which highlights the key role of IAA in promoting ginseng rhizome elongation. In conclusion, IAA and KT play major roles in regulating TLCG and TQCG rhizome elongation and may interfere with SA, GA, and ZT to jointly regulate the phenotypic development of ginseng rhizomes.

### 3.4. Screening the Key Regulatory Factors Affecting the Phenotypic Plasticity of Ginseng Rhizomes

**Figure 6:**
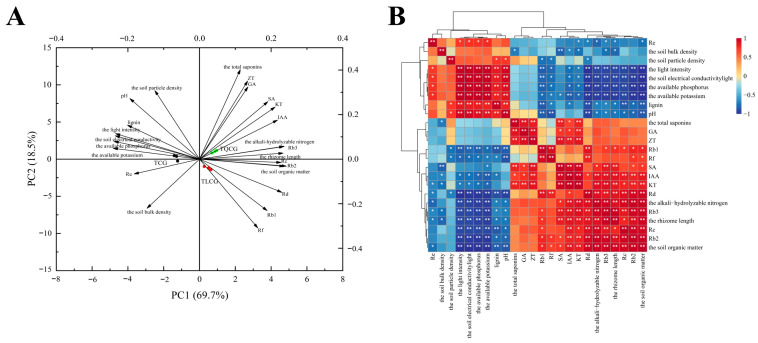
Principal component analysis and correlation analysis of ginseng rhizome length with ecological factors and metabolites. (**A**): principal component analysis; (**B**): correlation analysis. An asterisk represents the difference at the 0.05 level, meaning the difference is significant; the two asterisks represent a 0.01 level difference, meaning the difference is very significant.

To identify the primary regulatory factors impacting ginseng rhizome length, we evaluated a range of variables, including ecological factors (light intensity, soil particle density, soil bulk density, pH, conductivity, alkali-hydrolyzable nitrogen, organic matter, available potassium, and available phosphorus), saponin composition (monomeric saponins Re, Rf, Rb1, Rc, Rb2, Rb3, and Rd and total saponins), endogenous hormones (SA, IAA, GA, KT, ZT), and lignin. These variables exhibited significant differences across the TCG, TLCG, and TQCG plots, and their interactions with rhizome length were analyzed. Principal component analysis (PCA) (Figure 6A) revealed distinct differences between the first two principal components, PC1 and PC2. PC1 accounted for 69.7% of the total variance, while PC2 contributed to 18.5%, cumulatively explaining 88.2% of the variance. Notably, the TLCG and TQCG plots clustered together on the side of PC1 with minimal distance between them, indicating a high similarity in rhizome phenotype, growth environment, and metabolic components between these two habitats. Correlation analysis (Figure 6B) further elucidated the relationship between rhizome length and various factors. Habitat-wise, rhizome length showed a positive correlation with organic matter and alkali-hydrolyzable nitrogen (*p* < 0.01) but a negative correlation with available phosphorus, available potassium, light intensity, and electrical conductivity (*p* < 0.01), as well as with soil bulk density and pH (*p* < 0.05). The strongest negative correlations were observed with available potassium, available phosphorus, and light intensity, identifying them as key negative regulatory factors for rhizome length. In terms of metabolism, rhizome length positively correlated with saponins Rb2, Rb3, and Rd and hormones KT, IAA, and SA (*p* < 0.01), as well as with saponins Rb1 and Rc (*p* < 0.05). Conversely, a negative correlation was found with lignin content (*p* < 0.05).

### 3.5. Analysis of Key Genes’ Expression in Ginseng Rhizomes in Different Habitats

**Figure 7:**
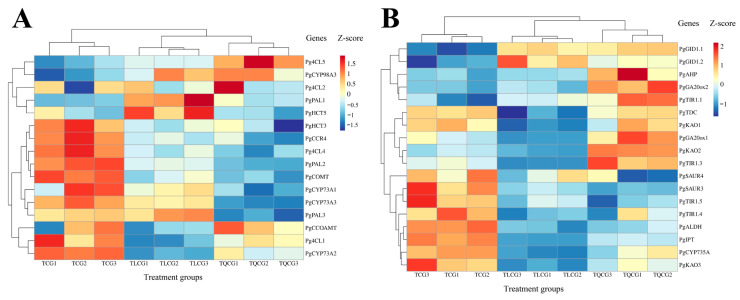
Changes in key genes’ expressions of ginseng rhizome in different habitats. (**A**): key genes in lignin biosynthesis pathway; (**B**): key genes in hormone synthesis and signal transduction pathway (The abscissa represents three treatment groups, and each treatment group has three biological repeats; the ordinate represents the names of key genes).

Our unpublished researches have established a close association between lignin biosynthesis, hormone synthesis, signal transduction pathways, and the phenotypic alterations observed in ginseng rhizomes. In this context, we analyzed the expression levels of pivotal genes involved in these pathways within the rhizomes collected from the TCG, TLCG, and TQCG plots (Figure 7). Our analysis identified 16 genes within the lignin synthesis pathway (Figure 7A). Among these, 11 genes showed significant down-regulation in the TLCG and TQCG samples and up-regulation in the TCG samples. These genes include phenylalanine ammonia-lyase (*PgPAL2, PgPAL3*), 4-coumaric acid coenzyme A ligase (*Pg4CL4, Pg4CL1*), cinnamoyl coenzyme A reductase (*PgCCR4*), shikimic acid/quinic acid hydroxy cinnamoyl transferase (*PgHCT3*), trans cinnamic acid 4-monooxygenase (*PgCYP73A1, PgCYP73A3*), caffeic acid 3-O-methyltransferase (*PgCOMT*), and caffeoyl-CoA-O-methyltransferase (*PgCCOAMT*). The down-regulation of these genes suggests a reduced propensity for lignin synthesis. This gene expression pattern correlates with the measured lignin content in the TLCG and TQCG rhizomes, reinforcing the notion that habitat changes influence ginseng rhizomes’ phenotypic variations by modulating lignin synthesis and accumulation.

Our analysis revealed that the gene expression profiles related to hormone synthesis and signal transduction in ginseng rhizomes from the TLCG and TQCG plots exhibit remarkable similarities, allowing these samples to be categorized together (Figure 7B). Specifically, we observed that the expression levels of genes related to gibberellin biosynthesis and signal transduction were significantly up-regulated in the TLCG and TQCG and significantly down-regulated in the TCG, including GA-20 oxidase (*PgGA20ox2*) and gibberellin receptor proteins (*PgGID1.1, PgGID1.2*). Conversely, genes involved in auxin signaling, such as the auxin transport inhibitory protein (*PgTIR1.4, PgTIR1.5*) and the auxin early response SAUR genes (*PgSAUR3, PgSAUR4*), were significantly down-regulated in the TLCG and TQCG rhizomes and significantly down-regulated in the TCG rhizomes. These findings indicate that the interplay between auxin and gibberellin signaling pathways is crucial for the phenotypic plasticity of ginseng rhizomes in response to changes in habitat. In essence, the crosstalk between auxin and gibberellin signaling pathways plays a pivotal role in adapting ginseng rhizome phenotypes to environmental variations.

### 3.6. Screening the Key Transcription Factors That Regulate the Phenotypic Difference in Ginseng Rhizomes

**Figure 8:**
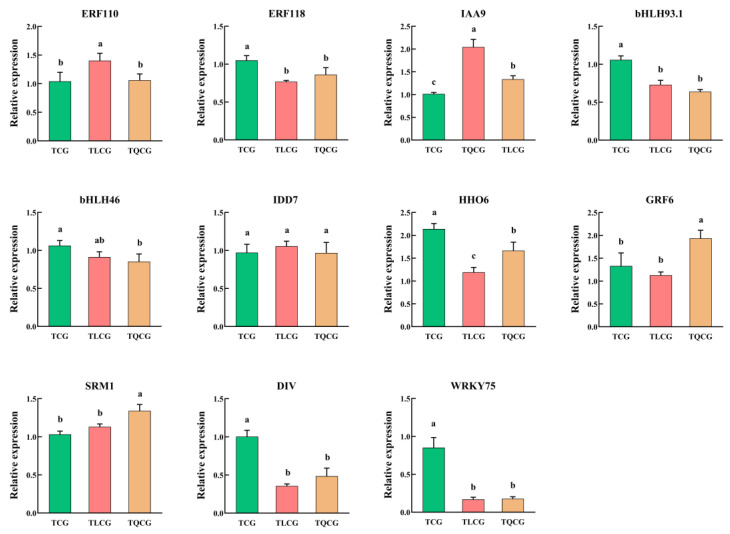
Changes in key transcription factors’ expressions of ginseng rhizomes in different habitats. Different lowercase letters indicate significant differences at the level of *p* < 0.05. Error bars depict standard deviations (±SD).

In our unpublished research, we identified 11 transcription factors potentially crucial in regulating the phenotype of ginseng rhizomes. To further understand their response to habitat changes, we measured the expression levels of these transcription factors in rhizomes from the TCG, TLCG, and TQCG plots (Figure 8). Our findings revealed that the expression levels of transcription factors *PgERF118, PgHHO6, PgbHLH93.1, PgDIV*, and *PgWRKY75* were significantly elevated in the TCG rhizomes compared to those in the TLCG and TQCG. Conversely, the expression level of transcription factor *PgIAA9* was notably lower in the TCG rhizomes than in the TLCG and TQCG. Overall, the expression profiles of transcription factors in the TLCG and TQCG were similar, yet distinct differences were observed when compared to the TCG, indicating their significant roles in adapting to habitat changes. Specifically, *PgHHO6* exhibited the highest expression level in the TCG, being 1.79 times and 1.28 times higher than in the TLCG and TQCG, respectively. Furthermore, *PgWRKY75* showed the most substantial difference across the habitats; the expression levels of *PgWRKY75* in the TCG rhizomes were 5.03 and 4.80 times that in the TLCG and TQCG, respectively, followed by *PgDIV* and *PgbHLH93.1*. Therefore, transcription factors *PgHHO6*, *PgWRKY75*, *PgbHLH93.1*, and *PgDIV* are implicated in actively responding to habitat changes and influencing the phenotypic adaptations of ginseng rhizomes.

### 3.7. Analysis of the Regulatory Network of Ecological Factors, Key Genes, and Transcription Factors

**Figure 9:**
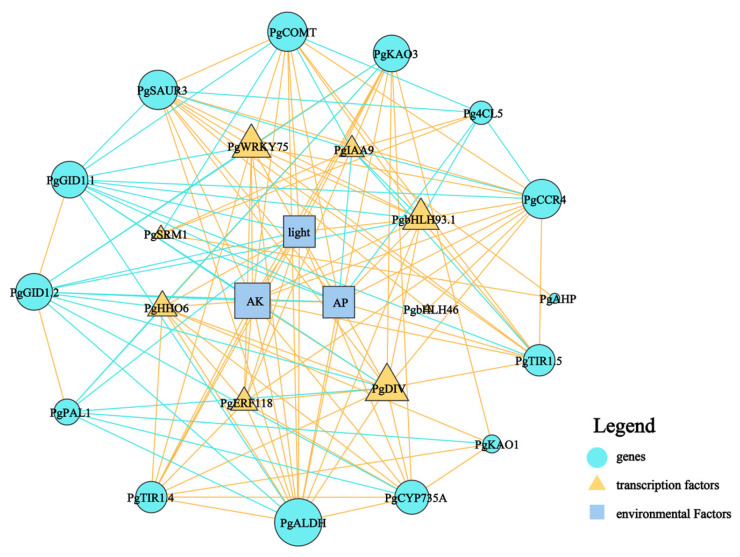
Correlation network analysis of dominant ecological factors and regulation of key genes and transcription factors in ginseng rhizomes. The size of the graph indicates the strength of the connection. The “blue line” indicates that there is a negative correlation between the two; The “orange line” indicates that there is a positive correlation between them.

The aboveground and underground parts of plants are closely connected through energy flow and information transfer, which jointly support the growth and development of plants and adapt to environmental changes. For example, the organic carbon produced by photosynthesis in the aboveground part of plants is transported to the underground part, which is beneficial to provide the energy needed for rhizome development. Meanwhile, some signal substances, such as hormones and ROS, are also transmitted in xylem and phloem and participate in regulating the growth and development of plant roots [37]. To further elucidate the internal mechanisms underlying the phenotypic plasticity of ginseng rhizomes in response to habitat changes, we constructed a correlation network diagram. This diagram included negative regulatory ecological factors such as light intensity (light), available potassium (AK), and available phosphorus (AP), alongside key enzyme genes for lignin synthesis, pivotal genes in hormone synthesis and signal transduction, and critical transcription factors (Figure 9). Our analysis revealed that AK, AP, and light were central nodes within the network, showing strong connections with other elements. Specifically, all three factors exhibited a positive correlation with the lignin synthesis gene *PgCOMT*, while AP and AK also showed a positive correlation with *PgCCR4*. In the realm of hormone transduction, AK, AP, and light were positively correlated with the auxin transport inhibitory proteins *PgTIR1.5* and *PgTIR1.4* but negatively correlated with the gibberellin receptor proteins *PgGID1.1* and *PgGID1.2*. Regarding transcription factors, AK, AP, and light demonstrated positive correlations with *PgWRKY75*, *PgDIV*, and *PgbHLH93.1*. Notably, *PgWRKY75*, *PgDIV*, and *PgbHLH93.1* exhibited similar effects, being positively correlated with *PgCCR4*, *PgCOMT*, *PgTIR1.5*, and *PgTIR1.4* and negatively correlated with *PgGID1.1* and *PgGID1.2*. In summary, the transcription factors *PgWRKY75*, *PgDIV*, and *PgbHLH93.1* play pivotal roles in the response of ginseng rhizomes to the different environments, such as AK, AP, and light.

## 4. Discussion

In our study, ginseng was transplanted into a farmland environment and two types of forest environments to assess the impact of habitat on rhizome phenotypes. The findings indicated that the forest environments were similar to each other but markedly distinct from the farmland environment. We evaluated the main ecological factors across these three settings. Notably, the light intensity in the farmland plot was significantly higher compared to the forest plots (Figure 1B). Similarly, the soil in the farmland plot contained considerably higher levels of available potassium and phosphorus than those found in the forest plots (Figure 1N,P). The most pronounced difference among these factors was in the available phosphorus content, which was 9.23 to 11.06 times higher in the farmland plot compared to the forest plots. The available phosphorus content in the forest plots was exceedingly low, indicating a state of phosphorus deficiency in the soil. Thus, light intensity and available phosphorus levels emerged as the primary ecological factors differentiating the farmland environment from the forest environment.

Current research indicates that plant roots exhibit considerable plasticity in low phosphorus environments, adapting by altering their shape and structure to improve phosphorus absorption and utilization [38,39]. This adaptation is a clear demonstration of plants’ ability to adjust to environmental challenges. However, the nature of root remodeling varies significantly across different plant species. For instance, rice plants respond to phosphorus scarcity by increasing the length and surface area of their root systems [40], whereas Arabidopsis thaliana responds by inhibiting taproot growth and encouraging the proliferation of root branches [41]. Moreover, light serves as a critical and complex ecological factor that not only fuels photosynthesis but also governs plant photomorphogenesis [42]. Previous studies on dicotyledonous plant-stem growth have shown that high-intensity blue light most effectively suppresses stem elongation [43], primarily by curtailing cell-wall elongation through the reduction of polysaccharide degradation, the promotion of lignin synthesis, and the reinforcement of cell-wall rigidity [44]. In shaded conditions, such as under a canopy, the reduced availability and altered quality of light trigger a range of shade avoidance responses in plants, including stem elongation, to capture more light [45,46]. Intriguingly, despite roots being shielded from direct light exposure, they are capable of responding to various signals that induce photomorphogenic reactions, thereby promoting root system elongation [17,47].

The ginseng rhizome, an underground stem resembling a root, is situated at the upper end of the root and directly contacts the soil. Our observations revealed that in forest environments, where light and phosphorus levels are low, the ginseng rhizome undergoes significant elongation, measuring 48.36% and 67.34% longer than those grown in farmland conditions (Figure 2B). This elongation mirrors the adaptive response of roots to environmental changes. Studies have shown that under the condition of low light, the photosynthesis of plants is limited, which may reduce the accumulation of carbon sources and then affect the growth of plants. On the other hand, under higher light intensity, the accumulation of ROS and lignin contents increased in plants. ROS, as an important signal molecule, interacts with JA-mediated signal transduction to regulate lignin biosynthesis in the elongation region cells of *Arabidopsis* seedlings [48]. Furthermore, we identified a close relationship between the elongation of the rhizome and the expansion of rhizome cells (Figure 2C,D). Correlation analysis indicated a strong negative correlation between rhizome length and both light intensity and available phosphorus content (Figure 6B), identifying these factors as crucial negative regulators of rhizome elongation. Consequently, we propose that to adapt to environments with limited light and phosphorus, ginseng rhizomes elongate to secure adequate light energy and nutrients. In essence, the elongation of the ginseng rhizome represents a growth tracking strategy in response to low light and phosphorus levels. This strategy reflects an adaptive mechanism where the plant follows environmental cues and exhibits alternative phenotypes to better align with its habitat, which belongs to a cross-species universal mechanism. In addition, based on the previous research (unpublished data from our research group), we screened out some differentially expressed genes related to the morphology of ginseng rhizomes, which were mainly involved in lignin biosynthesis, endogenous hormone biosynthesis, and signal transduction. In order to further explore the molecular mechanism of ginseng rhizomes adapting to the environment in this study, we took the differentially expressed genes in the above pathways as candidate genes.

The elongation of the ginseng rhizome is intricately linked to the elongation of rhizome cells (Figure 2), a process that entails the loosening and remodeling of cell walls to facilitate the necessary structural transformations for normal cell and organ morphogenesis [49,50]. Lignin, a primary constituent of the cell wall, plays a pivotal role in this context. High levels of lignin deposition stiffen the cell wall, thereby restricting its further expansion [22,23]. Research indicates that under shading stress, a reduction in lignin deposition within stem cells leads to an elongation of plant internodes [51], suggesting that lignin accumulation is inversely related to stem elongation. Despite the rhizome’s subterranean growth, the influence of lignin on its elongation mirrors that observed in stems. Compared with ginseng in farmland, the rhizomes of ginseng in the forest environments exhibited the lowest lignin content, and the expression of key enzyme genes *PgCCR4* and *PgCOMT* in lignin synthesis was significantly down-regulated (Figure 4 and Figure 7A). *RgCCR6* was also found to be the key gene affecting the lignin content of rhizomes in the study of *Rehmannia glutinosa* [52], which was similar to the conclusion of this study. Therefore, the decrease of lignin deposition in ginseng rhizome cells is helpful for ginseng rhizome elongation to adapt to the forest environment. Intriguingly, habitat changes also significantly impact the content of ginsenosides, ginseng’s main medicinal component. The challenging conditions of low light and low phosphorus found in forest environments notably enhance the levels of protopanaxadiol saponins and total saponins in the ginseng rhizome (Figure 3), thereby improving its internal quality. This correlation between rhizome morphology and internal quality underscores a unity, revealing that certain adverse conditions may actually be beneficial for the synthesis and accumulation of ginsenosides [53].

The spatial distribution and content changes of plant hormones are fundamental in controlling plant growth and development. The role of gibberellin in plant growth was initially highlighted through its ability to promote internode elongation in rice, thereby influencing plant stature via cell elongation and division [54]. Our research also found that the gibberellin content in the rhizome of ginseng increased with the increase of rhizome length under the forest environment (Figure 5C). Furthermore, we observed that elevated endogenous gibberellin contents enhanced the expression of gibberellin receptor proteins *PgGID1.1* and *PgGID1.2*, thereby activating gibberellin signaling pathways involved in rhizome elongation (Figure 7B). Similarly, indole-3-acetic acid (IAA) is the main active substance of auxin. Research indicates that auxin facilitates cell elongation and internode elongation in bamboo through the plant’s hormone signal pathway and amino acid synthesis pathway [55]. Additionally, auxin can acidify the extracellular environment via proton efflux, facilitating the loosening of hydrogen bonds between lignin molecules, thereby promoting cell-wall extension and regulating plant-cell and internode-tissue elongation [56,57]. This is similar to the results of this study. Compared with ginseng under farmland, we also found that the IAA content in the rhizomes of ginseng under forests increased significantly, and the auxin signal transduction genes *PgTIR1.5* and *PgTIR1.4* were activated (Figure 5B and Figure 7B). This may also be an important reason for the remarkable elongation of ginseng rhizomes under-forest. Interestingly, IAA and GA exhibit synergistic effects in promoting cell division and growth. For instance, IAA has been shown to induce internode elongation in peas by regulating the production of bioactive GA [58,59], while GA can influence internode elongation in Arabidopsis thaliana by modulating IAA synthesis and transport [60]. In conclusion, it shows that ginseng can adjust rhizome elongation through auxin and gibberellin contents in adaptation to habitat changes.

Transcription factors are proteins capable of binding to specific gene sequences [61]. They can operate singly or form complexes with other proteins to either enhance or inhibit the recruitment of RNA polymerase to specific genes, thereby regulating gene expression [62,63]. Notably, the bHLH family of transcription factors plays a crucial role in various aspects of plant development, including stress resistance, pathogen defense, and nutrient absorption [64]. Several bHLH transcription factors have been identified as key regulators of longitudinal cell elongation, contributing significantly to the structural formation of stems, roots and leaves, and exhibiting pronounced influence on phenotypic plasticity in response to light signals [64,65,66]. Similarly, specific WRKY and MYB transcription factors have shown heightened sensitivity to phosphorus deficiency, leading to morphological changes aimed at enhancing phosphorus content and uptake [67,68]. Our findings indicate significant differences in the expression levels of *PgWRKY75, PgDIV*, and *PgbHLH93.1* in ginseng rhizomes grown under forest conditions compared to those in farmland environments (Figure 8), with a notable correlation to available phosphorus and light intensity (Figure 9). Based on these observations, we hypothesize that *PgWRKY75*, *PgbHLH93.1*, and *PgDIV*, belonging to the MYB transcription factor family, are actively responsive to low-light and phosphorus environments. These factors play pivotal roles in regulating the elongation of ginseng rhizomes, demonstrating their critical function in plant adaptation to environmental stresses. Unfortunately, the environmental indicators measured in this study are limited, and other environmental indicators, such as air humidity, light quality, and soil microorganisms have not been measured and analyzed. Therefore, in future research, we will consider measuring more environmental factors and designing targeted ecological regulation experiments. In addition, the further analysis of plant hormones, especially auxin gradient, is also an important content to explore the phenotype-regulation mechanism of ginseng rhizomes.

## 5. Conclusions

This research demonstrates that the elongation of the ginseng rhizome is an adaptive growth strategy to low-light and phosphorus conditions. Transcription factors *PgWRKY75*, *PgDIV*, and *PgbHLH93.1* exhibit a dynamic response to changes in the habitat. The activation of auxin and gibberellin signal transduction pathways plays a pivotal role in this adaptation. Concurrently, the down-regulation of key lignin-synthesis enzyme genes *PgCOMT* and *PgCCR4* leads to a reduction in lignin deposition. This reduction is advantageous for the expansion of rhizome cells, ultimately facilitating the elongation of the ginseng rhizome. The findings from this study shed new light on the morphogenesis of ginseng, highlighting the complex interplay of hormonal signaling and transcriptional regulation in plant adaptation to environmental stresses. At the same time, it also provides a theoretical basis for optimizing the cultivation techniques of ginseng in different environments and cultivating high quality ginseng in a targeted manner.

## Data Availability

The datasets for this study are available in this manuscript and the Supplementary Materials.

## Supplementary Materials

The following supporting information can be downloaded at: https://www.mdpi.com/article/10.3390/cimb46110728/s1, Table S1. The primer sequence of differential genes. Table S2. Classification standard of soil organic matter and N, P, and K.
