# Supplementary material for: The Adaptive Mechanism of Ginseng Rhizomes in Response to Habitat Changes
Source: Curr Issues Mol Biol. 2024 Oct 30;46(11):12260–78. doi: 10.3390/cimb46110728 (PMC11593245; doi:10.3390/cimb46110728)
Supplement: Supplementary file 1 [file cimb-46-00728-s001.zip › cimb-3273931-supplementary.pdf]

Table S1. The primer sequence of differential genes.

Gene	Forward primer sequence	Reverse primer sequence
PgALDH	TGCTGACGCCCAAGCTAG	TCATGCATGCTCTCGCCC
PgTDC	TCCTGCAGCAACGGAACT	TCCACCACCCTGTCCTGT
PgKAO-1	CCCGGTTGGCCAAGTTCT	TTCACCGGTGCAGCAGTT
PgGA20ox-1	ACCTTAGGAACGGGGCCT	TGGACCGCCATTCGTTGT
PgIPT	AAGCTCGTTGTGGGACCG	GTTGCCATGGCCGTCTCT
PgCYP735A	TGAACCATGACCGTGCCC	TTGGTTGCATCCCCCGTG
PgTIR1-4	TGCAAGGGTCTCAAGCGG	TCCCGCTAGCAAACGCAA
PgSAUR-3	TTCCTCCCCCGCTCTCTC	GTGGTGGTGGTGCATGGA
PgSAUR-4	TCCGCACGCTAAAGCTCC	ATCGGGTGGTTCAGCAGC
PgAHP	AGCAGACACCAGGCAACC	CCAAAACCCCCTCGCAGT
PgGID1-1	CCCGGCTGCATTTGAGGA	AAAGCGTCGGCAATGTGC
PgGID1-2	AGCGTCAATGGTGGAGCC	AGCCTTCCCGCCTCTACA
PgPAL-1	GGAGTTGTGTGAGGGGGC	TGCGCCAAATCCTGTCGT
PgPAL-2	GACTAGGCGGCGAGACAC	CCTCCGAGAGCTCCACCT
Pg4CL-1	ACCCTGAGGCCACAGAGA	GCAGGGGCTACTTGGAAGC
Pg4CL-2	GTCTGGTGCTGCCCCATT	TTTGCAAACGCCAGGCAC
Pg4CL-4	TACTCCTCCGGGACCACG	CAACCTGTTGCGCCACAC
Pg4CL-5	CGTACTCCTCCGGGACCA	GACCTGTTGAGCCACGCT
PgCOMT	ACGCGCTACCGGATTCTG	TCTCTTTTCCCCCGGGGT
PgCYP73A-1	TTGAGGAGGCGGTTGCAG	CTCCCCGTTCAACGCCTT
PgCYP73A-2	CGCCGTCATCGTAGCCAT	CGGTGAGATTGCGGTGGT
PgCYP98A-3	AGTGGTGCCTCTGTTGTTCC	AAGCCCGACTCGTCCATG
PgHCT-3	CCGTGCCTTGGTCCCATT	TCTCCGCCTCCACGAAGA
PgCCOAMT	ATGGTTCCTTGGCTGCCC	GATCAACGGCCAGGGCTT
PgERF110	AAGAACAGGAGGCCACGC	TTCGCCGGACCTTTGACC
PgERF118	TCGAGGAAGCTCTTGCGT	CCACTCCAGAGCAACCCC
PgIAA9	GGTCAGGGCAATGCGTCT	ACGCCGGAGAGCTGTCTA
PgbHLH93-1	TTGAATGGGCAGGGGCAG	AGCATGGAAAGCCGTTCA
PgbHLH46	CCCCTTCCTGTGCCACTG	TCAGGTGTCAGTCGGGGT
PgIDD7	TGTCTGCAACGGCCTTGT	TTGGGCCTGCCATGCTAC
PgHHO6	TCGCTTCTCACTCCCGGA	GCAGCCTGCTGTTGTGATG
PgGRF6	GCCCCCGATTGGCTTCAT	CAAGGCAGGCAACGAGGA
PgSRM1	TGCAATTCTCCGCCGGTT	CAACCTTCTGCCACCGGT
PgDIV	ACATTACCCCCTCGGCCT	GGGATTCGGCCGAGCTAG
PgWRK75	ACGAAGGGAAGCACACACA	GGTAGGGAAGGGAGGGCA
PgKAO-2	AAGTGGCTGTTGGGCTCC	GGCCAGCCCAAATCACCT

PgKAO-3	ATGGGCAGACATGGGGGA	AACGCCTCCATCACCTGC
PgGA20ox-2	CCTGACCACGAAACGCCT	AGATGGCAAGGGGTTCGC
PgTIR1-1	TGCAAGGGTCTCAAGCGG	CCTTGTCACTCCCGCCAG
PgTIR1-3	GCCCACCGGTCCAAGTTT	CGCTGGATTGACGGTGGA
PgTIR1-5	CTGGTGGCAAGGTGTCGT	TGAGGCGCTTGCACAAGA
PgCCR-4	AGTAGGGGCCCTGACGAG	TTGCCTCTTCCCACGCTG
PgHCT-5	CAAGGGTGCAGTCGGTGT	TCCCGCATATTCACCGCT
PgCYP73A-3	GAGGCAAAGCTTGGGGGT	GCCAACCCACCTTCCTCC
PgPAL-3	GACTAGGCGGCGAGACAC	CCTCCGAGAGCTCCACCT

Table S2. Classification standard of soil organic matter and N, P and K.

Projects/Grade	Level 1	Level 2	Level 3	Level 4	Level 5	Level 6
Organic matter (%)	＞4	3-4	2-3	1-2	0.6-1	＜0.6
Total nitrogen (g/kg）	＞2	1.5-2	1-1.5	0.75-1	0.5-0.75	＜0.5
Alkali-hydrolyzable nitrogen (mg/kg)	＞150	120-150	90-120	60-90	30-60	＜30
Total phosphorus (g/kg）	＞2	1.5-2	1-1.5	0.75-1	0.5-0.75	＜0.5
Available phosphorus (mg/kg)	＞40	20-40	10-20	5-10	3-5	＜3
Total potassium (g/kg）	＞20	15-20	10-15	5-10	3-5	＜3
Available potassium (mg/kg)	＞200	150-200	100-150	50-100	30-50	＜30
